# Microarray Analysis of Siberian Ginseng Cyclic Somatic Embryogenesis Culture Systems Provides Insight into Molecular Mechanisms of Embryogenic Cell Cluster Generation

**DOI:** 10.1371/journal.pone.0094959

**Published:** 2014-04-17

**Authors:** Chenguang Zhou, Likun Liu, Chenghao Li

**Affiliations:** 1 State Key Laboratory of Forest Genetics and Tree Breeding, Northeast Forestry University, Harbin, China; 2 Department of Medical Biotechnology, College of Biomedical Science, Kangwon National University, Chuncheon, South Korea; Wuhan University, China

## Abstract

Four systems of cyclic somatic embryogenesis of Siberian ginseng (*Eleutherococcus senticosus* Maxim) were used to study the mechanism of embryonic cell cluster generation. The first, direct somatic embryo induction (DSEI), generates secondary embryos directly from the primary somatic embryos; the second, direct embryogenic cell cluster induction (DEC)), induces embryogenic cell clusters directly from somatic embryos in agar medium. Subsequently, we found that when DEC-derived somatic embryos are transferred to suspension culture or a bioreactor culture, only somatic embryos are induced, and embryogenic cell clusters cannot form. Therefore, these new lines were named DEC cultured by liquid medium (ECS) and DEC cultured by bioreactor (ECB), respectively. Transmission electron microscopy showed that DEC epidermal cells contained a variety of inclusions, distinct from other lines. A cDNA library of DEC was constructed, and 1,948 gene clusters were obtained and used as probes. RNA was prepared from somatic embryos from each of the four lines and hybridized to a microarray. In DEC, 7 genes were specifically upregulated compared with the other three lines, and 4 genes were downregulated. *EsXTH1* and *EsPLT1*, which were among the genes upregulated in DEC, were cloned using the rapid amplification of cDNA ends (RACE). Real-time quantitative PCR showed *EsXTH1* was more highly expressed in DEC than in other lines throughout the culture cycle, and *EsPLT1* expression in DEC increased as culture duration increased, but remained at a low expression level in other lines. These results suggest that *EsXTH1* and *EsPLT1* may be the essential genes that play important roles during the induction of embryogenic cell clusters.

## Introduction

Somatic embryos are embryos derived from somatic cells that are capable of developing into young plantlets via a series of morphological changes, a process that closely resembles that of zygotic embryos [1]. Direct somatic embryogenesis (DSE) is a way of developing young plantlets by direct differentiation from explants without an intervening callus phase. During DSE, somatic cells turn into embryogenic cells, and then enter into a differentiation phase. Sometimes, the embryogenic cells do not differentiate directly, but proliferate for a period of time before the proliferated cells start to differentiate. These cells are called the embryogenic cell clusters, and the process is considered a type of DSE because of the direct generation of embryogenic cells. Studies of DSE and embryogenic cell cluster formation have been prevalent [2], [3], and cell proliferation is more conductive to increasing the heritability coefficient. One culture cycle can yield a large number of somatic embryos through embryogenic cell cluster process.

Studies identified several genes that play regulatory roles either in specific phases of embryogenesis or during the entire process [4], [5], such as *BABY BOOM* (*BBM*), which plays a more general role in maintaining cells in an undifferentiated state and which proteins belong to the AP2 subfamily [6]; *WUSCHEL* (*WUS*), which promotes somatic embryo development in seedlings when ectopically expressed [7]; *AGAMOUS-LIKE15* (*AGL15*), which promotes somatic embryogenesis in part by controlling ethylene biosynthesis and response [8]; and *LEAFY COTYLEDON* (*LEC*), which is essential for induction of somatic embryo development [9]. During embryogenesis, specific remodeling of the cell wall is a crucial process. Xyloglucan endotransglucosylases/hydrolases (XTHs) possess both xyloglucan endotransglucosylase (XET) and xyloglucan endohydrolase (XEH) activities and provide the enzymatic activities responsible for cell wall plasticity. Two *XTH*-related genes showed a high level of expression during SE induction in *Cucumis sativus* [10].

Several recent experiments applying expressed sequence tag, microarray, and transcriptional profile analysis were performed to define the molecular events for somatic embryogenesis (SE) [8], [11]–[16]. Although many SE studies used genetic techniques and obtained a large number of genes, few reports screened differentially expressed genes between embryogenic cell cluster and direct somatic embryo induction. Previous studies mainly targeted the differences between two SE pathways: DSE and ISE (indirect somatic embryogenesis; indirect differentiation after a callus phase) [5].

Siberian ginseng (*Eleutherococcus senticosus* Maxim) plantlets have been obtained through somatic embryogenesis using semisolid and suspension cultures both using DSE and ISE [17], [18]. Siberian ginseng SEs has been successfully produced in bioreactors [19], [20]. In this study, we compared the cellular structures and molecular mechanisms between four lines of repetitive somatic embryos of Siberian ginseng that were obtained by different inductive conditions. One condition was direct induction of secondary embryos from primary somatic embryos, and the other was DEC, where embryogenic cell clusters were induced from primary somatic embryos in agar medium. Our previous study found that somatic embryos, but not embryogenic cell clusters, were induced directly when DEC-derived somatic embryos were transferred into shaken flasks or a bioreactor to grow [20]. Ultrastructural observation showed significant differences of epidermal cells among four lines of Siberian ginseng embryos developed in this study. We report here the screening, isolation, and functional prediction of candidate genes using EST, microarray, and differential expression analysis in controlling embryogenic cell cluster induction in Siberian ginseng.

## Materials and Methods

### Plant materials and growth conditions

Seeds of Siberian ginseng were stratified in moist sand at 15°C for 6 months; dehiscent seeds were chosen as the culture material. After removing the coat, seeds were sterilized in 70% ethyl alcohol for 30 sec followed by 1% NaClO for 10 min, then rinsed 5 times with sterile water. Sterilized seeds were transferred onto 1/3 MS (Murashige and Skoog) medium with 1% sucrose. Cultures were maintained under white fluorescent light [photosynthetic photon flux density (PPFD): 40 µmol·m^−2^· s^−1^] and long day conditions (16 h light/8 h dark).

For direct somatic embryo induction (DSEI), seeds whose cotyledon had been exposed for 1–3 days were incubated at 40°C for 5 days, then transferred to plant growth regulator (PGR)-free 1/3 MS medium and cultured for 3 months without medium exchange. For direct embryogenic cell cluster induction (DEC), somatic embryos were transferred to 1/3 MS medium with 1 mg·L^−1^ 2,4-D (2,4-Dichlorophenoxyacetic acid); this medium possesses the ability to continuously generate embryogenic cell clusters. Embryogenic cell clusters were transferred onto 1/3 MS medium without PGR for somatic embryo development, and mature embryos eventually formed [20].

### Induction of secondary somatic embryos

Somatic embryos from DEC and DSEI were used for generating secondary somatic embryos. The primary somatic embryos were cultured on 1/3 MS medium with 1% sucrose without PGR for secondary embryo induction. Secondary somatic embryos arising from the somatic embryos were used for further proliferation by repeated subculture onto fresh medium of the same composition.

Secondary somatic embryos from DEC were transferred into 1/3 MS liquid medium without PGR for ECS and ECB into shaken flask suspension cultures [20] and bioreactor cultures [20], respectively. Embryo explants in both instruments were collected on the fifth day of the culture cycle.

### Transmission electron microscope (TEM) observation

Somatic embryo explants generated by DEC, DSEI, ECS and ECB were collected on the 5th day. The samples were pre-fixed in 3% glutaraldehyde in phosphate buffer [pH 7.4] for at least 3 hours at 4°C. After rinsing 3 times with phosphate buffer, samples were fixed with 1% osmium tetroxide for 1–2 hours, dehydrated in an increasing concentration series of acetone, and embedded in epoxy resin. Ultrathin sections were obtained with an ultramicrotome (Leica Ultracut UCT) and contrasted with uranyl acetate and lead citrate. Image collection was performed with a transmission electron microscope (FEI/Philips TCNAI G2) at 50 kV.

### cDNA library construction

Total RNA from DEC embryos on the fifth day of culture was extracted by the SDS method. mRNA was isolated using the PolyATtract Isolation System III Kit (Promega, Madison, WI, USA), following the manufacturer's instructions. A 3 M NaAc aliquot was added to the mRNA at 1/10 of the total volume, and an equal volume of isopropanol was added to precipitate the mRNA at −20°C overnight. Following centrifugation at 15,000 × g for 30 min, the mRNA was rinsed with 70% ethanol, then 100% ethanol, dried, and dissolved in RNase-free water.

Five micrograms of mRNA was used to construct the reference library using the cDNA Library Construction Kit and following the manufacturer's instructions (Takara, Otsu, Japan). Ten microliters of bacteria containing the unamplified library was diluted 10-, 10^2^-, and 10^3^-fold in succession with SOC liquid medium, 10 µL of diluted bacteria liquid were then plated onto LB medium agar plates containing 30 mg/L of Ampicillin (Amp); plates were incubated for 12 h at 37°C, and the titer of the unamplified library was measured. Clones from the cDNA library were randomly selected for sequencing, and expressed sequence tags (ESTs) were used for bioinformatic analysis. Initially, we assembled EST sequences using the Phred/Phrap/Consed package. For annotation, the assembled consensus sequences and singletons were used as queries in searches of the Genebank Nt, Genebank Nr, and SWISSPORT databases performed using BLASTN and BLASTX with an *E*-value cut-off of 10^−10^. Singletons were annotated and functionally classified by comparison with sequences in the COG database.

### Microarray production

Probes were designed based on the contigs of the Siberian ginseng cDNA library. cDNA from DEC, ECS, ECB and DSEI on the fifth day of culture was hybridized with probes. Two hybridization replicates were performed.

The tissue was homogenized in liquid nitrogen using a mortar and pestle, and total RNA was prepared using the SDS method. After precipitation, Qiagen RNeasy Kit (Qiagen, Valencia, CA, USA) was used for purification. RNA from each sample was reverse transcribed into aminoallyl-labeled cDNA, using 2 µg of total RNA primed with 5 µg of T7 promoter primer, and 200 U of M-MLV reverse transcriptase (Takara). The cDNA was purified using Qiagen RNeasy Mini kit (Qiagen). The cDNA (4 µg) was concentrated to 6.6 µL, mixed with 10 µL DMSO, and added to 3.4 µL of 0.3 M NaHCO_3_ (pH 9.0). The mixture was added into fluorescent dye (Cy3). Excess dye was removed using the Qiagen RNeasy Mini kit (Qiagen). Then, 11 µL of 10× Blocking Agent and 2.2 µL of 25× Fragmentation Buffer were added to the purified Cy3 labeled targets to a final volume of 55 µL, and 55 µL of 2× GEx hybridization buffer was added for fragmentation. The mixture (100 µL) was put on the slide for rolling hybridization at 65°C for 17 h. After hybridization, the slide was washed at 37°C. The slides were dried, and then scanned using an Agilent scanner.

### Quantitative RT-PCR

On the fifth day of culture, total RNA from DEC and DSEI was prepared using the SDS method. All of the qRT-PCR reactions, including the validation of upregulated and downregulated genes in the microarray assays, were carried out on RQ1 RNase-free DNase (Promega). cDNA synthesis from total RNA was performed using the PrimeScript RT reagents Kit (Takara) according to the manufacturer's instructions. Assays were performed using SYBR Premix Ex Taq II (Takara) kit for quantification. Primers were designed as 17–25 nt oligomers with Tm 60–65 to amplify a product 80–150 bp long (Table S1). Quantification was performed by 2^ΔΔC (t)^ using *actin* and *tubulin* as housekeeping genes for normalization.

### *EsPLT1* and *EsXTH1* gene cloning

Total RNA of DEC was extracted using the SDS method. 5′-RACE-Ready and 3′ -RACE-Ready cDNA synthesis were performed using the SMART RACE cDNA Amplification Kit (Takara) and following the manufacturer's instructions. The full length *EsPLT1* was then cloned by 5′/3′-rapid amplification of cDNA ends (5′/3′-RACE). Primers were designed based on EST sequences using the RACE instructions and Primer Premier 5.0. RACE products were cloned into TA plasmids using pGEM-T and pGEM-T Easy Vector Systems (Promega). After sequencing, the 5′-RACE product and 3′-RACE product were aligned and spliced based on the overlap by using CONTIG software. We then designed a pair of primers based on the obtained sequence corresponding to the nucleotide at position ‘ATG’ and ‘TGA’. The PCR product was then cloned into pGEM-T Vector and sequenced to obtain the full-length gene sequence. *EsXTH1* cloning was the same as the above procedure.

## Results

### The induction of four embryogenic culture lines

**Figure 1:**
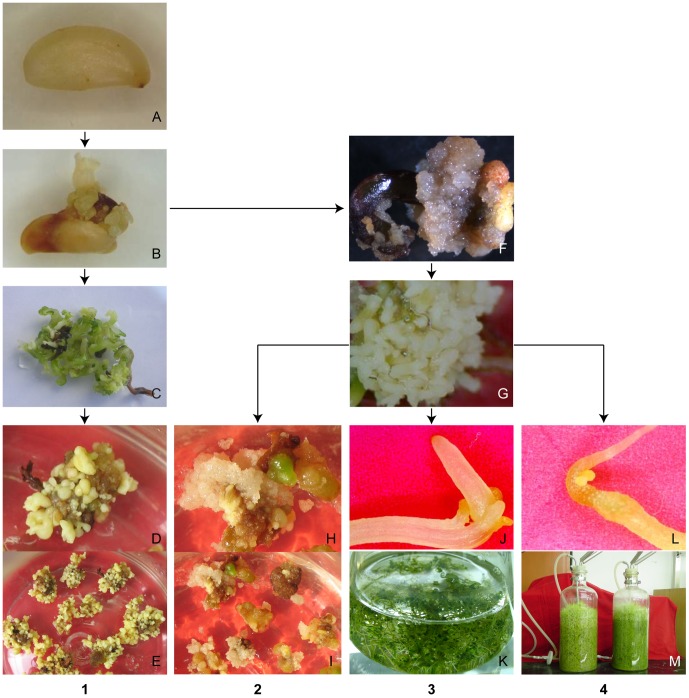
Schematic diagram of the process used to generate four lines (DEC, DSEI, ECS and ECB) of somatic embryos. **A** Mature seed, **B** somatic embryos were induced from mature seed directly through heat-stressed culture on hormone-free 1/3 MS medium, **C** secondary embryos were developed directly after transfer somatic embryos to the original culture environment cultured on hormone-free medium for 3–4 weeks, **D** somatic embryos were developed when secondary embryos were subcultured on hormone-free medium, **E** somatic embryos, **F** embryogenic cell clusters were induced from somatic embryos through culture on 1/3 MS medium with 1 mg·L^−1^ 2,4-D, **G** mature embryos were developed when embryogenic cell clusters were cultured on hormone-free medium, **H** embryogenic cell clusters were generated when embryos were cultured on 1/3 MS medium with 1 mg·L^−1^ 2,4-D, **I** embryogenic cell clusters, **J** secondary embryos were developed directly when mature embryos (DEC line) were transferred into 1/3 MS liquid medium without PGR for suspension culture, **K** secondary embryos in suspension culture, **L** secondary embryos were developed directly when mature embryos (DEC line) were transferred into 1/3 MS liquid medium without PGR for bioreactor culture, **M** secondary embryos in bioreactor. **1.** DSEI process. **2.** DEC process **3.** ECS process **4.** ECB process.

Mature seeds (Figure 1A) stopped developing upon treatment with high temperature; at the same time, the surface of the endosperm and the edge of cotyledon became slightly brown and somatic embryos developed directly (Figure 1B). These somatic embryos developed into secondary embryos directly after transfer to the original culture environment for DSEI (Figure 1C). When these somatic embryos isolated, they again gave rise to new small somatic embryos in a repetitive manner (Figure 1D, E). Additionally, visible secondary embryos occurred within 7 days on the surface of the original somatic embryos.

When somatic embryos were cultured on medium containing 1 mg/L^−1^ 2,4-D, friable embryogenic cell clusters formed from the surface of primary somatic embryos (Figure 1F). DEC developed mature embryos upon culture on PGR-free medium (Figure 1G). We selected one lines of secondary somatic embryos generated embryogenic cell clusters again in a repetitive manner on PGR-free medium (Figure 1H, I). Visible embryos cells also occurred within 7 days from original somatic embryos. Embryogenic capacity could maintenance for more than 5 years by repeated secondary embryogenesis.

When DEC lines were transferred into liquid medium in a shaken flask suspension culture (ECS) and bioreactor culture (ECB), the somatic embryos developed into secondary embryos directly (Figure 1J, K and L, M) [20]. The secondary embryos failed to generate embryogenic cell clusters either in shaken flasks or bioreactor cultures, and did not generate embryogenic cell clusters after being transferred again to induction medium (data not shown).

### Observation of somatic embryo epidermal cells by TEM

**Figure 2:**
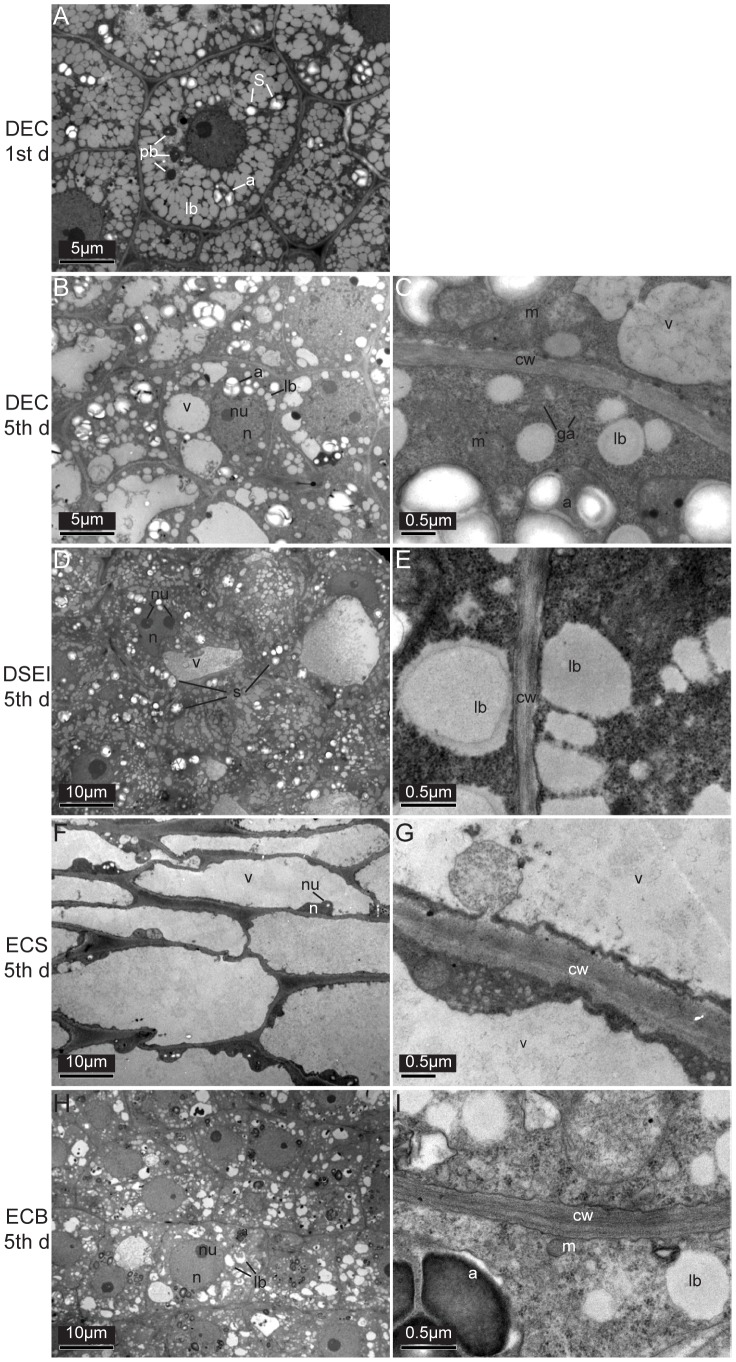
TEM observation of epidermal cells in DEC, DSEI, ECS and ECB of Siberian ginseng somatic embryos. Epidermal cells in DEC on the first day of culture (A); epidermal cells in DEC (B, C), DSEI (D, E), ECS (F, G) and ECB (H, I) on the 5th days of culture. *a* amyloplast, *cw* cell wall, *g* glyoxysome, *Ga* Golgi apparatus, *lb* lipid body, *m* mitochondria, *n* nucleus, *nu* nucleous, *pb* protein body, *s* starch grain, *v* vacuole.

On the first day of cultivation, TEM of DEC showed epidermal cells completely filled with amyloplasts, protein bodies, and numerous uncoalesced lipid bodies (Figure 2A).

On the 5th day of cultivation, TEM of epidermal cells of DEC, DSEI, ECS and ECB showed significant differences; DEC showed protoplasm with a large nucleus, prominent nucleolus, numerous lipid bodies, mitochondria, small, irregular-shaped vacuoles, and glyoxysomes next to the lipid bodies. Accumulation of starch grains was observed in amyloplasts with dense stroma (Figure 2B, C). The cell wall was thicker, and the intercellular gap was larger than other lines of cells.

Protoplasm of DSEI contained large nuclei, prominent nucleoli, nonuniform vacuoles, numerous lipid bodies, and amyloplasts (Figure 2D). Electron dense plate structures were observed in the cytoplasm (Figure 2E). The cell wall of DSEI was unclear and had an irregular edge.

TEM of ECS showed that the nucleus was pushed into the edge of the cell wall by a central vacuole (Figure 2F), and few cell inclusions were observed (Figure 2G).

Epidermal cells of ECB were rich in inclusions and comprised lipid bodies, amyloplasts, protein bodies, mitochondria, and glyoxysomes next to the lipid bodies (Figure 2H, I). The nucleus and nucleolus were clearly observed. Cell walls were clear, but irregular, and the intercellular space was narrowed compared with other cell lines.

### cDNA library construction

Siberian ginseng embryonic cell cluster library was generated from somatic embryos of DEC with an unamplified library titer of 2.3×10^6^ pfu·mL^−1^. The range of inserted fragment length was 500–2000 bp, and the average length was 960 bp. Randomly sequenced clones from 5376 cDNA clones and 3691 ESTs were obtained. After removing vector sequences and sequences of inferior quality, 3331 sequences with effective length greater than 100 bp were obtained (library accession # LIBEST_028309). Then, 2415 unigenes that comprised 497 contigs and 1918 singletons were obtained after assembly of the ESTs. The length of unigenes was 300–1200 bp.

**Figure 3:**
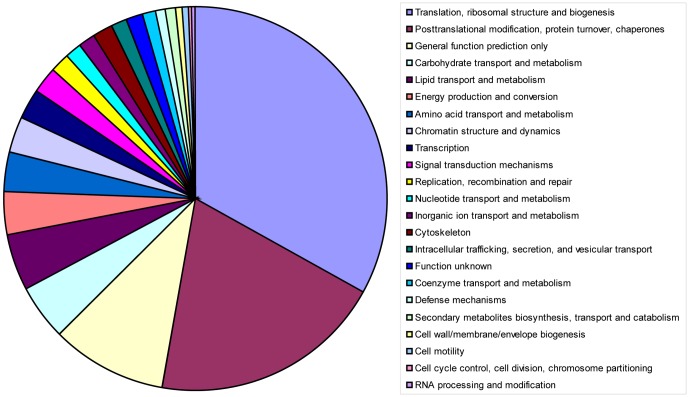
Pie charts showing functional categories assigned using COG. 490 clusters predicted to encode characterized proteins classified into 23 different functional classifications.

The unigenes were used for annotation through BLASTN and BLASTX searches of the NCBI Nt, NCBI Nr, SWISSPROT and KEGG databases. In total, 1570, 1657, 1188 and 1629 unigenes matched these database entries, respectively. Comparison of unigenes against the COG database resulted in the tentative annotation of 490 unigenes including 7 unigenes of unknown function (Table 1). Sequences encoding proteins putatively involved in translation and ribosomal structure and biogenesis comprised the largest functional group (33.06%; Figure 3). The sequences related to posttranslational modification, protein turnover, and chaperones comprised the second largest category (19.59%), and proteins related to lipid transport and metabolism comprised a considerable portion (4.49%).

**Table 1:** **Differentially regulated genes in DEC compared with DSEI, ECS and ECB.**

GenBank Accession number	Fold change			Up-Down Regulation	BLASTX sequence similarity (accession number)	E value
	DEC vs. DSEI	DEC vs. ECS	DEC vs. ECB			
JZ513132	8.00	3.14	2.30	Up	PLT2, *A. thaliana* (NM_103997.3)	2.00E-09
JZ513081	8.62	3.29	2.42	Up	HBP-1b (c1)-like, *V. vinifera* (XM_002281994.2)	3.00E-10
JZ512520	3.45	2.61	2.13	Up	XTH, *V. vinifera* (XM_003633163.1)	2.00E-21
JZ512170	5.88	2.28	4.77	Up	Phosphate transporter, *P. trichocarpa* (XM_002306809.1)	8.00E-51
JZ513874	4.07	4.11	2.12	Up	No significant similarity found	
JZ512623	2.06	2.15	3.64	Up	DREB2, *S. tuberosum* (JN125858.1)	5.00E-58
JZ513828	2.51	4.26	4.69	Up	No significant similarity found	2.00E-04
JZ511890	44.91	2.14	3.16	Down	CASP-like protein 1, *Panax ginseng* (Q20BM9)	3.00E-30
JZ514138	2.31	6.33	2.38	Down	SDR, *R. communis* (XM_002509770.1)	4.00E-63
JZ514908	36.95	2.60	3.59	Down	Thaumatin-like protein, *V. vinifera* (XM_002265816.1)	6.00E-23
JZ514859	26.63	2.27	2.19	Down	No significant similarity found	

### Microarray analysis

To find the differentially expressed genes related to development of embryogenic cell clusters, fluorescent probes were synthesized based on the 1948 contigs described above (LIBEST_028309). Total RNA samples were extracted from DEC, DSEI, ECS and ECB lines, and hybridized with array.

The list of expressed genes was significantly different among DEC, DSEI, ECS and ECB lines (Table S2, 3, 4). It was found that 552 transcripts were upregulated and 335 transcripts were downregulated in the DEC line compared with DSEI (Table S2A, B), 40 upregulated and 50 downregulated compared with ECS (Table S3A, B), and 55 upregulated and 44 downregulated compared with ECB (Table S4A, B).

To screen for major genes related to the generation of embryogenic cell clusters, the DEC line was compared with other three lines together, and 7 upregulated genes and 4 downregulated genes were found (Table 1). The functions of these genes were predicted using gene ontology analysis to determine in which biological process and cellular component they functioned. In the DEC line, the upregulated genes were involved in stem cell activity, stress resistance, and regeneration of cell wall. In contrast, the commonly upregulated genes in DSEI, ECS and ECB, were involved in plant protective effect, redox sensor mechanisms, and biotic and abiotic stress.

### Validation of the expression pattern of genes detected by microarray analysis

**Figure 4:**
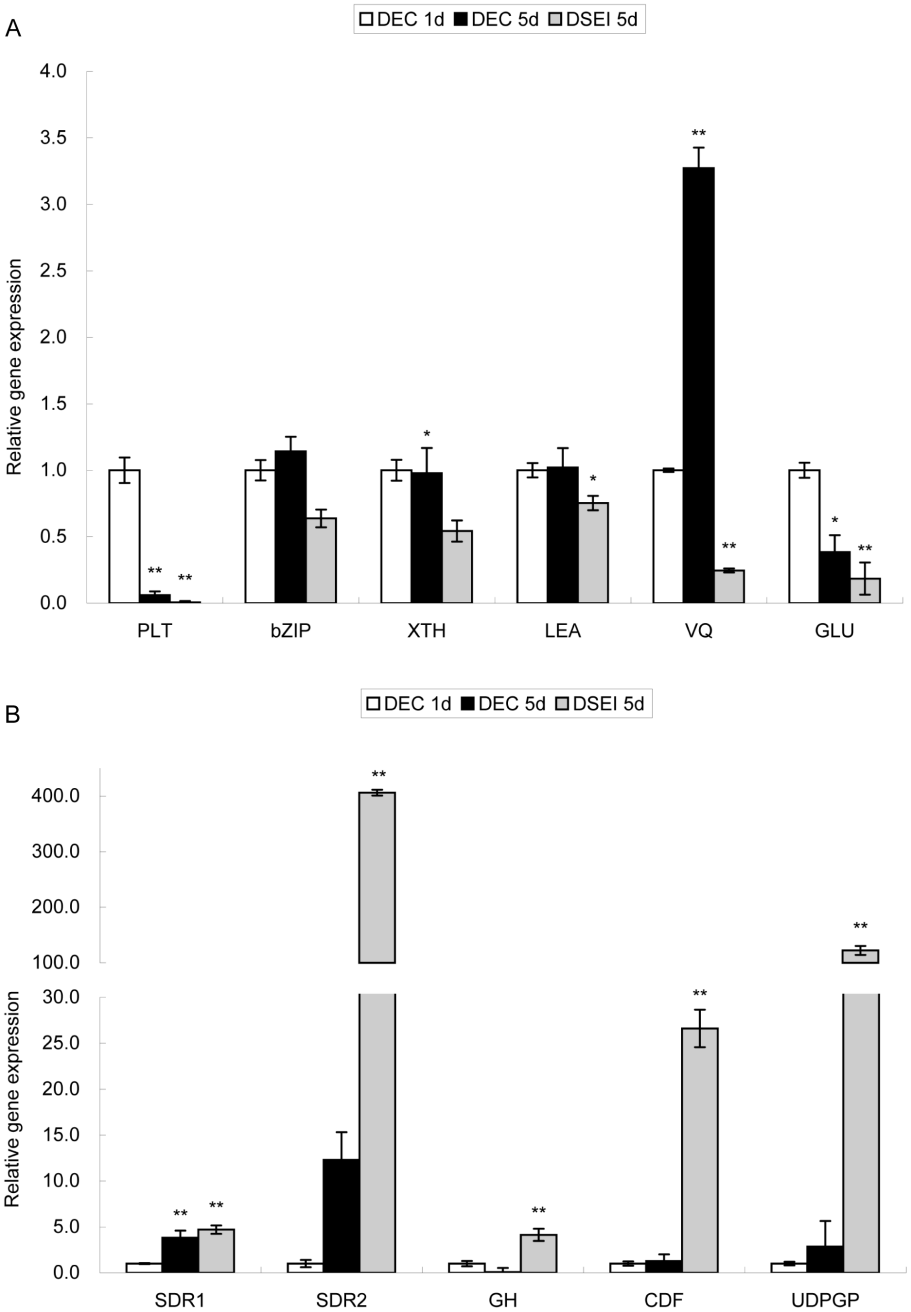
Validation of microarray chip data using quantitative RT-PCR. **A** The expression of upregulated genes. **B** The expression of downregulated genes. Results are an average of three biological replicates ± standard error. Genes were normalized using *actin* and *tubulin* as housekeeping genes. Genes expression in DEC on the first day of culture were used as control. Statistically significant differences are indicated (* P<0.05 and ** P<0.01 by two-tailed t-test and one-way ANOVA).

To confirm the data of the microarray analysis, quantitative real-time PCR was used to determine the expression level of several selected genes that were differentially expressed between the DEC and DSEI lines (Table 2). *PLT*, *bZIP* and *LEA* stress resistance related genes, *XTH* cell wall remodeling enzyme related gene, and *GLU* cell wall protein related gene were all more highly expressed in DEC than in DSEI (Figure 4A). The response to changing environmental conditions for plants with a VQ motif was almost 7-fold higher in DEC than DSEI. The validation of the microarray data by quantitative PCR showed that the results obtained by both methods were consistent with each other.

**Table 2:** **Differentially regulated genes in DEC compared with DSEI.**

GenBank Accession number	Experimental name	Fold change	Up-Down Regulation	BLASTX sequence similarity (accession number)	E value
JZ513132	PLT	8.00	Up	PLT2, *A. thaliana* (NM_103997.3)	2.00E-09
JZ513081	bZIP	8.62	Up	HBP-1b (c1)-like, *V. vinifera* (XM_002281994.2)	3.00E-10
JZ512520	XTH	3.45	Up	XTH, *V. vinifera* (XM_003633163.1)	2.00E-21
JZ511852	LEA	3.02	Up	LEA1, *A. hypogaea* (ADQ91833)	1.00E-33
JZ512706	VQ	2.66	Up	Structural constituent of cell wall, *R. communis* (XP_002513957)	9.00E-08
JZ514398	GLU	3.19	Up	Endo-1,3/1,4-beta-D-glucanase, *V. vinifera* (XP_002275697)	2.00E-44
JZ514138	SDR1	2.31	Down	SDR, *R. communis* (XM_002509770.1)	4.00E-63
JZ512158	SDR2	431.34	Down	Glucose and ribitol dehydrogenase, *M. truncatula* (XP_003591095)	4.00E-33
JZ512411	GH	12.42	Down	Glucosidase precursor, *R. communis* (XM_002510658.1)	3.00E-12
JZ514265	CDF	103.26	Down	Cell growth defect factor-2, *A. thaliana* (NM148494.2)	1.00E-19
JZ514152	UDPGP	330.78	Down	UDP-glucose pyrophosphorylase, *Zea mays* (DAA62613)	1.00E-15

Genes that were downregulated in the DEC line and had an extremely large difference in expression were chosen to be validated using quantitative PCR (Table 2). Two *SDR* short-chain dehydrogenases/reductases genes, the *GH* antifungal protein related gene (*GH*, Glucan endo-1,3-beta-glucosdase), the *CDF* cell growth defect factor related gene, and a major form of the nucleoside diphosphoglucose gene UDP-glucose pyrophosphorylase (*UGPGP*) were all upregulated in DSEI compared with DEC. *SDR2* and *GH* were upregulated more than 300-fold in DSEI (Table 2). This result was consistent with the microarray chip. Relative expression of other genes was higher in DSEI than in DEC (Figure 4B), consistent with the microarray chip.

### Cloning of full-length cDNA encoding *EsXTH1* and *EsPLT1*

**Figure 5:**
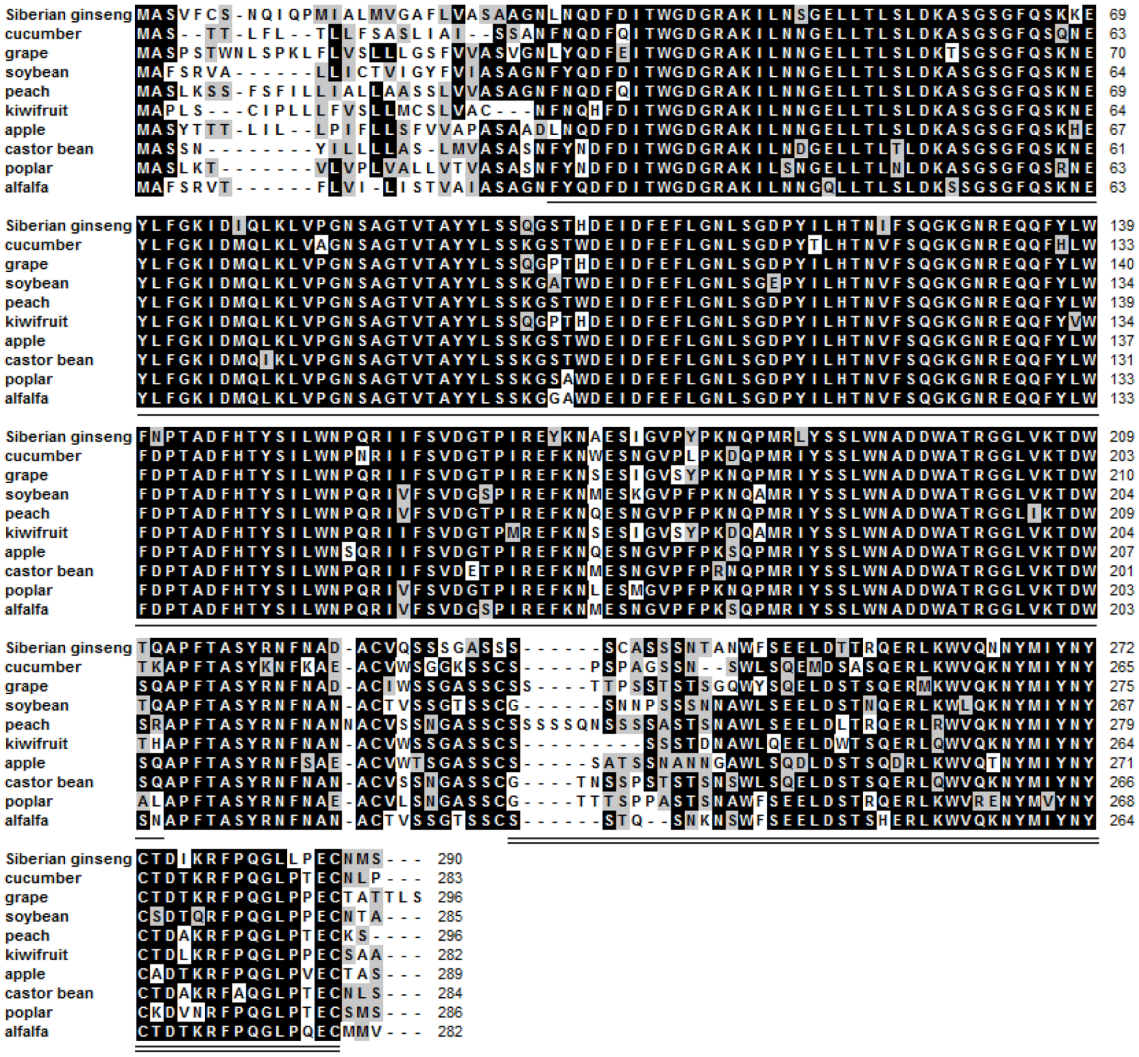
Amino acid sequence alignment of *EsXTH1* with XTH from other plant species. Black represents 100% homology; gray represents ≥ 50% homology. Amino acid XTH from cucumber (*Cucumis sativus*, XM 004165650.1), grape (*Vitis vinifera*, XM 002270146.2), soybean (*Glycine max*, XM 003542108.1), peach (*Prunus persica*, KB 638913.1), kiwifruit (*Actinidia hemsleyana*, EU 494954.1), apple (*Malus domestica*, EU 494967.1), castor bean (*Ricinus communis*, XM 002526182.1), poplar (*Populus trichocarpa*, XM 002324444.1), and alfalfa (*Medicago truncatula*, XM 003625289.1) revealed the existence of conserved functional domains of Glyco_hydro_16 (*lined section*) and XET_C (*double lined section*).

*EsXTH1* (accession no. KF660542), which was upregulated in DEC, was cloned using RACE and sequenced. An 1101-bp cDNA containing 873-bp of coding sequence was obtained, and the sequence was predicted to encode a protein of 290 amino acids (Figure S1A). Conserved domains were found in *EsXTH1* by aligning the cDNA sequence to other plant XTH sequences (Figure 5). According to Simple Modular Architecture Research Tool (SMART) (http://smart.embl-heidelberg.de/), amino acids 30-211 aligned to glycosyl hydrolases family 16 (E-value 1.2e-62), and amino acids 236–287 aligned to the C-terminus of xyloglucan endotransglycosylase (XET) (E-value 3e-22); the conserved domains that were recognized are underlined in Figure 4B.

**Figure 6:**
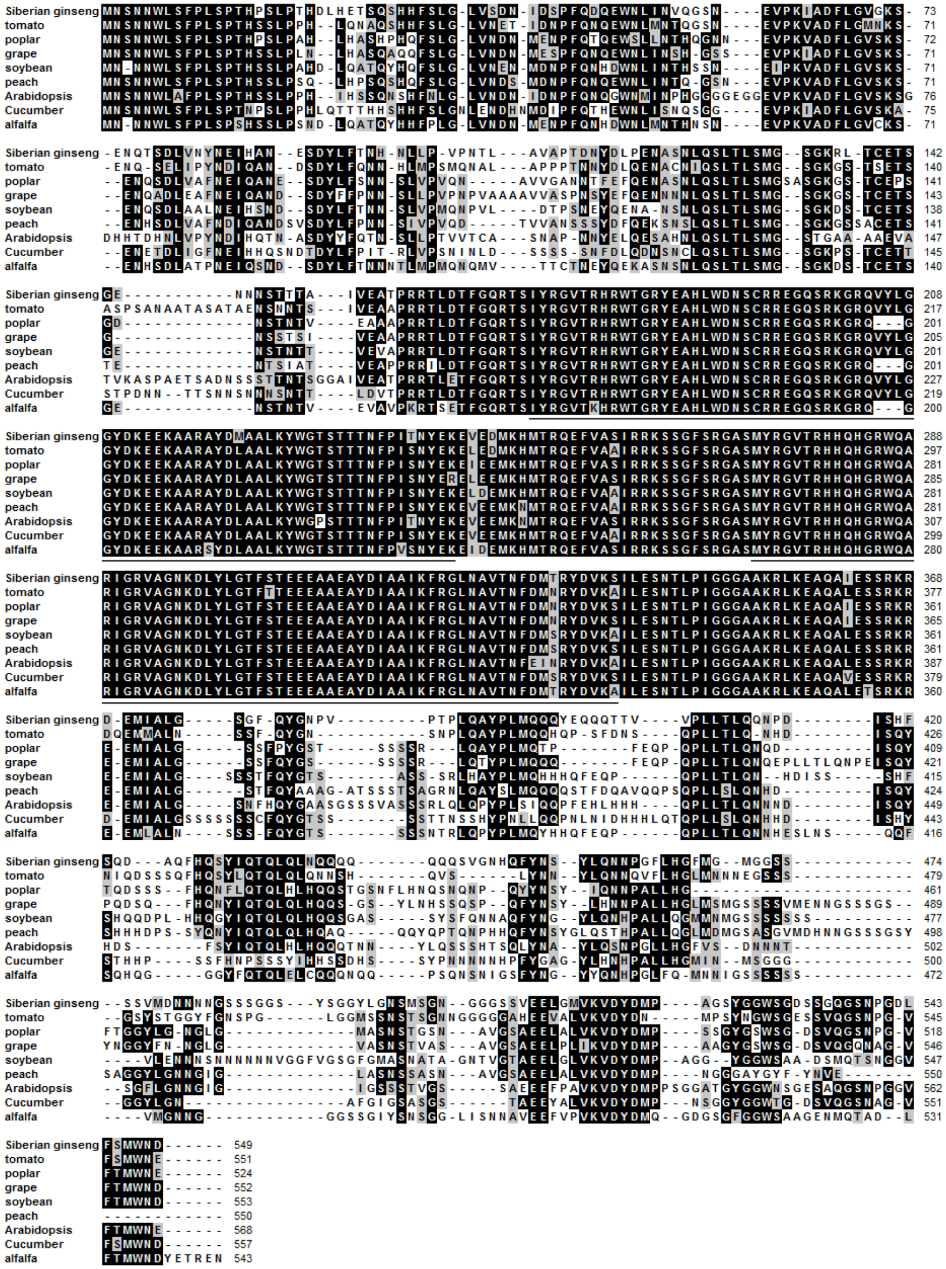
Amino acid sequence alignment of *EsPLT1* with PLT from other plant species. Black represents 100% homology; gray represents ≥ 50% homology. Amino acid sequences of PLT from tomato (*Solanum lycopersicum*, XM 004250817.1), poplar (*P. trichocarpa*, XM 002303852.1), grape (*V. vinifera*, XM 002285503.1), soybean (*G. max*, XM 003539373.1), peach (*Prunus persica*, KB639090.1), *Arabidopsis* (*A. thaliana*, NM 103997.3), cucumber (*Cucumis sativus*, XM 004142079.1), and alfalfa (*M. truncatula*, XM 003606710.1) revealed the existence of conserved functional domains of AP2 (*lined section*).

*EsPLT1* (accession no. KF660541), which was also upregulated in DEC, was cloned using RACE. The cDNA was sequenced, and a 2026-bp cDNA sequence was obtained that contained a 1650-bp coding sequence, and was predicted to encode a 549-amino acid protein in length (Figure S1B). The cDNA was aligned to other plant PLTs (Figure 6). These include the following domains underlined in Figure 6, which are characteristic of an AP2 subfamily that belongs to the AP2/EREBP family: amino acids 171–243 (E-value 1.02e-29) and amino acids 273–337 (E-value 8.63e-33) constitute the AP2 DNA-binding domain (according to http://smart.embl-heidelberg.de/).

### Expression of *EsXTH1* and *EsPLT1* mRNA

**Figure 7:**
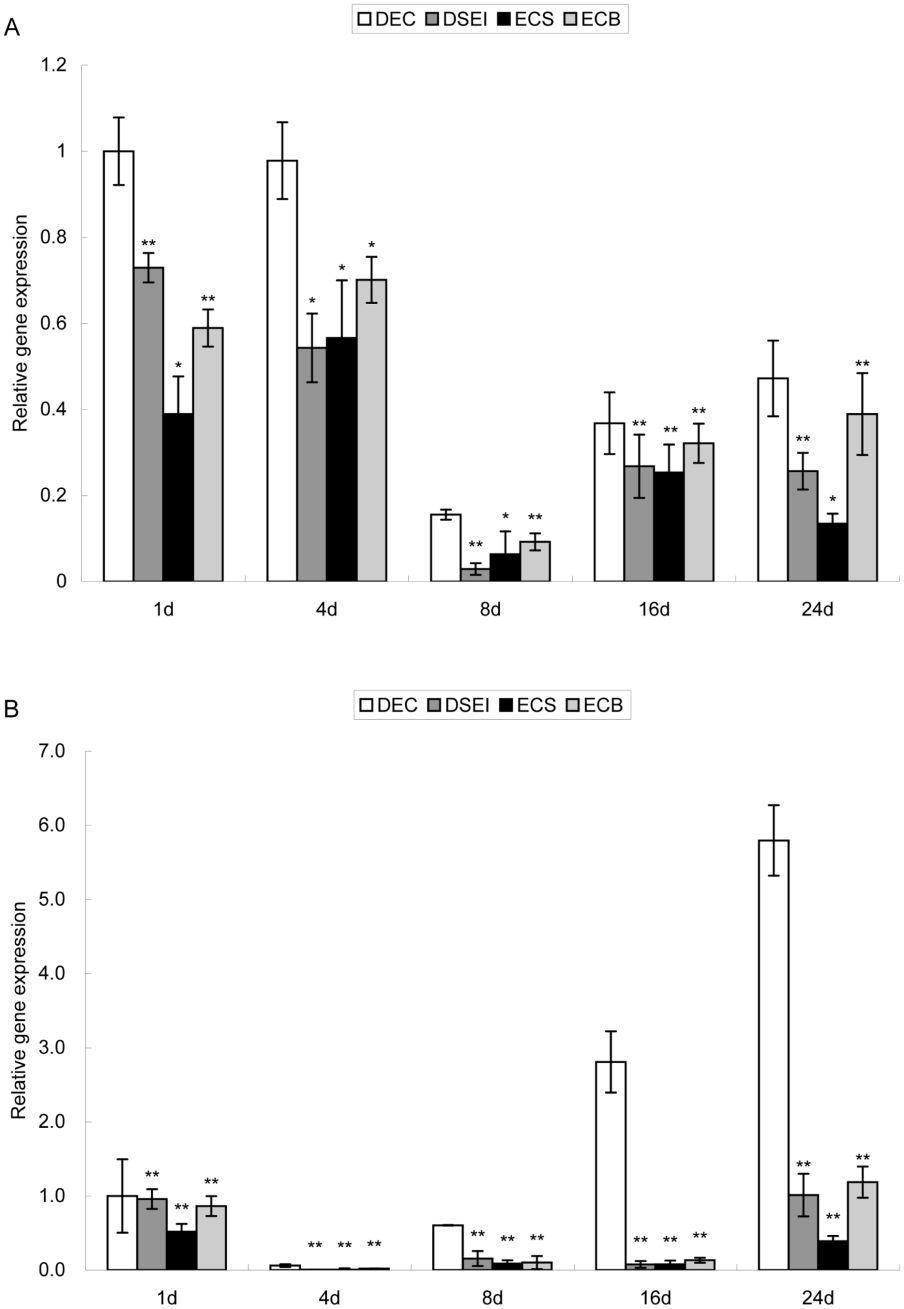
Expression of *EsXTH1* (A) and *EsPLT1* (B) genes in DEC, DSEI, ECS and ECB. Results were an average of three biological replicates ± standard error. Genes were normalized using *actin* and *tubulin* as housekeeping genes. Gene expression on the first day of culture was used as the control. Statistically significant differences are indicated (* P<0.05 and ** P<0.01 by two-tailed t-test and one-way ANOVA).

To better understand the gene expression pattern of *EsXTH1* and *EsPLT1*, relative expression of these two genes in the DEC and DSEI lines was measured over a culture cycle. *EsXTH1* was more highly expressed in DEC than in other lines throughout the duration of the culture cycle (Figure 7A). The change in overall gene expression in ECS, ECB and DSEI lines was approximately the same, for example, gene expression in ECB was higher than in ECS and DSEI and lower than in DEC throughout the culture cycle. Gene expression in DSEI and ECS was almost the same. *EsPLT1* in DEC had increased expression with increased culture duration, but remained at a low expression level in other lines (Figure 7B). At the beginning of the culture stage, *EsPLT1* was expressed at a very low level in all four lines, but at the following stage, expression increased sharply only in DEC.

## Discussion

Four repetitive somatic embryogenesis lines of *E. senticosus* were obtained through different inductive conditions. The different characteristics of DEC, DSEI, ECS and ECB lines allow for different utilities of each line, however, the ability of the DEC line to produce a large number of embryogenic cell clusters is more beneficial for propagation of Siberian ginseng.

TEM was used to observe the ultrastructural differences in epidermal cells among DEC, DSEI, ECS and ECB lines. It was found that DEC epidermal cells had dense cytoplasm, prominent nuclei, notable amounts of reserve compounds, and slightly large mitochondria, as verified in embryogenic cells in other species [21], [22]. It is generally known that reserve compounds are enriched in the initial stage of SE. Our study found only lipids and proteins in the epidermal cell of Siberian ginseng. Before the start of the formation of the protuberances, proteins and lipids became smaller and were partially or totally consumed. This time coincides with the period where epidermal cells acquire meristematic characteristics [23]. Starch grains were substituted for proteins and lipids in the initial stages of the embryogenic process. A high amount of starch has been observed during this time in different species [21], [24], [25]. Ultrastructural characteristics were notably different in DEC compared with ECS and DSEI; similarities were observed between DEC and ECB. Abundant inclusions in ECB, as in DEC, may be the reason for the rapid growth of somatic embryos; therefore, this culture method may be useful for industrial production of Siberian ginseng. Plasmodesmata were not observed in all embryos; only in DEC lines were more regularly spherical cells observed, a characteristic of embryogenic cell formation [26]. This regular spherical shape may be what caused the cells in the DEC line to separate more easily and generate the embryogenic cell clusters. Golgi apparati were found near the cell walls in the DEC line, which provided polysaccharide for thickening the cell wall. In the early stage of embryogenic cells, signals have to be transferred through plasmodesmata for embryogenic cells to acquire the information for their differentiation and division [27]. The disappearance of plasmodesmata and the thickening of the cell wall provided a physiological isolation between embryogenic cells and other surrounding cells, releasing the embryogenic cells from the control of the surrounding cells and allowing them to have totipotency [21], [26].

The significant differences in ultrastructure suggested that the involvement of various physiological pathways was different among DEC, DSEI, ECS and ECB in Siberian ginseng, and the pathway regulating somatic embryo development in ECS and ECB changed when DEC lines were transferred into either liquid medium or bioreactor cultures, respectively. During construction of the Siberian ginseng cDNA library (LIBEST_028309), 1948 expressed sequence tags (ESTs) were obtained. A considerable portion of these ESTs corresponded to genes involved in translation, ribosomal structure and biogenesis, posttranslational modification, protein turnover, and chaperones. These biological processes indicated that gene reprogramming occurred [26], [28]. Genes involved in lipid transport and metabolism were also highly expressed (Figure 3). We found robust lipid bodies present at the initial development of somatic embryos (Figure 2A), which became smaller and less numerous as development progressed (Figure 2B). The increased activity of genes related to lipid transport and metabolism could help explain this ultrastructural change.

During embryogenesis, the cell wall remodeling process is a crucial step. Polysaccharides, cell wall components, need to be disassembled and remodeled because of cell enlargement [29]. Xyloglucan is one type of polysaccharide present, and is the predominate hemicellulose in the cell walls of most dicotyledons. It forms a network with cellulose that strengthens the cell wall. Xyloglucan endotransglucosylases/hydrolases (XTHs), belonging to sixteenth family of glycoside hydrolases, take part in the remodeling step, and are essential for this process [30]. It is worth mentioning that XET (most XTHs possess this activity) was identified as the enzyme responsible for splitting xyloglucan chains and linking the newly-generated reducing end to the non-reducing ends of another xyloglucan chain, resulting in the wall-loosening required for plant cell expansion [30], [31]. Two *XTH*-related genes were identified as having increased expression during SE in *Cucumis sativus* [10]. One *XTH*-related gene named *EsXTH1* (accession no. KF660541) was found in Siberian ginseng, and was expressed more highly in the DEC line than in the ECS, ECB, and DSEI lines that do not generate embryogenic cell clusters. Among the upregulated genes in DEC were many genes related to cell wall modification. This finding implies the active role of cell wall modifying enzymes during Siberian ginseng development, particularly during embryogenesis. Our result suggests that cell wall remodeling is more likely to occur in DEC, potentially explaining why embryogenic cells develop more easily in that line.

The genes belonging to the AP2/ERF family have roles in undifferentiated cell proliferation and differentiation of stem cell niches within meristems [32], for example, *BBM* encodes an AP2 domain transcription factor and expressed in developing embryos and seeds, maintains cells in an undifferentiated state [6]. Besides, *PLT1* and *PLT2* genes of *Arabidopsis* were identified to encode AP2-family putative transcription factors. These genes are essential for quiescent center (QC) specification and stem cell activity [33]. It was found that the transcribed activity of *PLT* genes is dependent on auxin accumulation and auxin response transcription factors. Conceptually, the embryogenic cell is similar to a stem cell. Both possess the abilities of cell division and cell totipotency. In the initial stage of SE, certain responsive cells have the potential to activate genes involved in generating embryogenic cells [5]. Auxin may be a factor that mediates the signal transduction cascade to activate those certain responsive cells. This embryogenesis process is similar to the generation of stem cells. In this study, an *AP2* gene that was upregulated in DEC of Siberian ginseng showed considerable similarity compared with *PLT* genes of other species (Figure 6). In comparing the relative gene expression of the *AP2* gene between DEC and DSEI of Siberian ginseng, expression was observed to be higher in DEC than in DSEI throughout the cultivation cycle (Figure 7B). Interestingly, the expression of *PLT* genes in DEC gradually increased over the duration of cultivation. In the same way, two *PLT* genes described in the *Arabidopsis* root have been shown to maintain the activity of stem cells [33], and subsequent research showed promoter activity and PLT activities are largely additive and dosage-dependent [34]. In this study, *EsPLT1* was expressed throughout cultivation of DEC, increasingly and gradually; in contrast, this phenomenon did not occur in DSEI, ECS, and ECB. Meanwhile, embryogenic cell clusters increased continually and exponentially app:addword:exponentiallyin quantity during cultivation. Therefore, we conclude that the *EsPLT1*, through its continually increasing level, gives DEC the ability to generate embryogenic cells. These results also demonstrated that embryogenic cells were identical to stem cells.

Embryogenic cell cluster induction is more beneficial for SE because of the rapid development and massive somatic embryos. It is worth researching on a molecular level to identify key genes involved in this process. This study suggested that *EsXTH1* and *EsPLT1* might be the essential genes that provide embryogenic ability for generation of embryogenic cell cluster in Siberian ginseng.

## Supporting Information

Figure S1 **The nucleotide and amino acid sequences of** ***EsXTH1*** **and** ***EsPLT1*** **.** **A** showed conserved functional domains of Glyco_hydro_16 (*gray section*) and XET_C (*lined section*). **B** showed conserved functional domains of AP2 (*gray section*).
(DOC)

Table S1 **Primer sequences information.**
(XLS)

Table S2 **Upregulated (A) and downregulated (B) genes of DEC compared with DSEI.**
(XLS)

Table S3 **Upregulated (A) and downregulated (B) genes of DEC compared with ECS.**
(XLS)

Table S4 **Upregulated (A) and downregulated (B) genes of DEC compared with ECB.**
(XLS)

## References

[1] Dodeman VL, Ducreux G, Kreis M (1997) Zygotic embryogenesis versus somatic embryogenesis. J Exp Bot 48: 1493–1509.

[2] Nomura K, Komamine A (1985) Identification and isolation of single cells that produce somatic embryos at a high frequency in a carrot suspension culture. Plant Physiol 79: 988–991. 16664558 10.1104/pp.79.4.988 PMC1075012

[3] Monja-Mio KM, Robert ML (2013) Direct somatic embryogenesis of *Agave fourcroydes* Lem, through thin cell layer culture. In Vitro Cell Dev-Pl 49: 541–549.

[4] Jenik PD, Gillmor CS, Lukowitz W (2007) Embryonic patterning in *Arabidopsis thaliana* . Annu Rev Cell Dev Bi 23: 207–236. 10.1146/annurev.cellbio.22.011105.102609 17539754

[5] Yang XY, Zhang XL (2010) Regulation of somatic embryogenesis in higher plants. Crit Rev Plant Sci 29: 36–57.

[6] Boutilier K, Offringa R, Sharma VK, Kieft H, Ouellet T, et al (2002) Ectopic expression of BABY BOOM triggers a conversion from vegetative to embryonic growth. Plant Cell 14: 1737–1749. 12172019 10.1105/tpc.001941 PMC151462

[7] Zuo J, Niu QW, Frugis G, Chua NH (2002) The *WUSCHEL* gene promotes vegetative-to-embryonic transition in *Arabidopsis* . Plant J 30: 349–359. 12000682 10.1046/j.1365-313x.2002.01289.x

[8] Zheng Q, Zheng Y, Perry SE (2013) *AGAMOUS-Like15* promotes somatic embryogenesis in *Arabidopsis* and soybean in part by the control of ethylene biosynthesis and response. Plant Physiol 161: 2113–2127. 23457229 10.1104/pp.113.216275 PMC3613480

[9] Gaj MD, Zhang SB, Harada JJ, Lemaux PG (2005) Leafy cotyledon genes are essential for induction of somatic embryogenesis of *Arabidopsis* . Planta 222: 977–988. 16034595 10.1007/s00425-005-0041-y

[10] Malinowski R, Filipecki M, Tagashira N, Wisniewska A, Gaj P, et al (2004) Xyloglucan endotransglucosylase/hydrolase genes in cucumber (*Cucumis sativus*) - differential expression during somatic embryogenesis. Physiol Plantarum 120: 678–685. 10.1111/j.0031-9317.2004.0289.x 15032830

[11] Yang XY, Zhang XL, Yuan DJ, Jin FY, Zhang YC, et al (2012) Transcript profiling reveals complex auxin signaling pathway and transcription regulation involved in dedifferentiation and redifferentiation during somatic embryogenesis in cotton. BMC Plant Biol 12: 110. 22817809 10.1186/1471-2229-12-110 PMC3483692

[12] Xu ZZ, Zhang CJ, Zhang XY, Liu CL, Wu ZX, et al (2013) Transcriptome profiling reveals auxin and cytokinin regulating somatic embryogenesis in different sister lines of cotton cultivar CCRI24. J Integr Plant Biol 55: 631–642. 23710882 10.1111/jipb.12073

[13] Gliwicka M, Nowak K, Balazadeh S, Mueller-Roeber B, Gaj MD (2013) Extensive modulation of the transcription factor transcriptome during somatic embryogenesis in *Arabidopsis thaliana* . Plos One 8: e69261. 23874927 10.1371/journal.pone.0069261 PMC3714258

[14] Zhang JH, Zhang SG, Han SY, Wu T, Li XM, et al (2012) Genome-wide identification of microRNAs in larch and stage-specific modulation of 11 conserved microRNAs and their targets during somatic embryogenesis. Planta 236: 647–657. 22526500 10.1007/s00425-012-1643-9

[15] Yang XY, Wang LC, Yuan DJ, Lindsey K, Zhang XL (2013) Small RNA and degradome sequencing reveal complex miRNA regulation during cotton somatic embryogenesis. J Exp Bot 64: 1521–1536. 23382553 10.1093/jxb/ert013 PMC3617824

[16] Lin YL, Lai ZX (2013) Comparative analysis reveals dynamic changes in miRNAs and their targets and expression during somatic embryogenesis in longan (*Dimocarpus longan* Lour.). Plos One 8: e60337. 23593197 10.1371/journal.pone.0060337 PMC3623967

[17] Choi YE, Kim JW, Yoon ES (1999) High frequency of plant production via somatic embryogenesis from callus or cell suspension cultures in *Elutherococcus senticosus* . Ann Bot 83: 309–314.

[18] Choi YE, Yang DC, Yoon ES (1999) Rapid propagation of *Eleuterococcus senticosus* via direct somatic embryogenesis from explants of germinating zygotic embryos. Plant Cell Tiss Org Cult 58: 93–97.

[19] Choi YE, Jeong JH (2002) Dormancy induction of somatic embryos of Siberian ginseng by high sucrose concentrations enhances the conservation of hydrated artificial seeds and dehydration resistance. Plant Cell Rep 20: 1112–1116.

[20] Yang JL, Zhou CG, Liu LH, Jia Du, Yin ZY, et al (2012) High conversion frequency of germinated somatic embryos of Siberian ginseng (*Eleutherococcus senticosus* Maxim) using a bubble column bioreactor. Plant Cell Tiss and Org Cult 110: 289–298.

[21] Canhoto JM, Mesquita JF, Cruz GS (1996) Ultrastructural changes in cotyledons of *Pineapple Guava* (Myrtaceae) during somatic embryogenesis. Ann Bot 78: 513–521.

[22] Blehová A, Bobák M, Šamaj J, Hlinková E (2010) Changes in the formation of an extracellular matrix surface network during early stages of indirect somatic embryogenesis in *Drosera spathulata* . Acta Bot Hung 52: 23–33.

[23] San-José MC, Corredoira E, Martínez MT, Vidal N, Valladares S, et al (2010) Shoot apex explants for induction of somatic embryogenesis in mature *Quercus robur* L. Trees. Plant Cell Rep 29: 661–671. 20376670 10.1007/s00299-010-0852-6

[24] Pinto G, Silva S, Neves L, Araujo C, Santos C (2010) Histocytological changes and reserve accumulation during somatic embryogenesis in *Eucalyptus globulus* . Trees 24: 763–769.

[25] Rocha DI, Vieira LM, Tanaka FAO, da Silva LC, Otoni WC (2012) Somatic embryogenesis of a wild passion fruit species *Passiflora cincinnata* Masters: histocytological and histochemical evidences. Protoplasma 249: 747–758. 21927886 10.1007/s00709-011-0318-x

[26] Fehér A, Pasternak TP, Dudits D (2003) Transition of somatic plant cells to an embryogenic state. Plant Cell Tiss Org Cul 74: 201–228.

[27] Ehlers K, Binding H, Kollmann R (1999) The formation of symplasmic domains by plugging of plasmodesmata: a general event in plant morphogenesis? Protoplasma 209: 181–192.

[28] De Jong AJ, Schmidt EDL, de Vries SC (1993) Early events in higher-plant embryogenesis. Plant Mol Biol 22: 367–377. 8507837 10.1007/BF00014943

[29] Cosgrove DJ (2000) Loosening of plant cell walls by expansins. Nature 407: 321–326. 11014181 10.1038/35030000

[30] Thompson JE, Fry SC (2001) Restructuring of wall-bound xyloglucan by transglycosylation in living plant cells. Plant J 26: 23–24. 11359607 10.1046/j.1365-313x.2001.01005.x

[31] Fry SC, Smith RC, Renwick KF, Martin DJ, Hodge SK, et al (1992) Xyloglucan endotransglycosylase, a new wall-loosening enzyme-activity from plants. Biochem J 282: 821–828. 1554366 10.1042/bj2820821 PMC1130861

[32] Nole-Wilson S, Tranby TL, Krizek BA (2005) *AINTEGUMENTA-like* (*AIL*) genes are expressed in young tissues and may specify meristematic or division-competent states. Plant Mol Biol 57: 613–628. 15988559 10.1007/s11103-005-0955-6

[33] Aida M, Beis D, Heidstra R, Willemsen V, Blilou I, et al (2004) The *PLETHORA* genes mediate patterning of the *Arabidopsis* root stem cell niche. Cell 119: 109–120. 15454085 10.1016/j.cell.2004.09.018

[34] Galinha C, Hofhuis H, Luijten M, Willemsen V, Blilou I, et al (2007) PLETHORA proteins as does-dependent master regulators of *Arabidopsis* root development. Nature 449: 1053–1057. 17960244 10.1038/nature06206

