# Supplementary material for: Microarray Analysis of Siberian Ginseng Cyclic Somatic Embryogenesis Culture Systems Provides Insight into Molecular Mechanisms of Embryogenic Cell Cluster Generation
Source: PLoS One. 2014 Apr 17;9(4):e94959. doi: 10.1371/journal.pone.0094959 (PMC3990593; doi:10.1371/journal.pone.0094959)
Supplement: Figure S1 — The nucleotide and amino acid sequences of EsXTH1 and EsPLT1 . A showed conserved functional domains of Glyco_hydro_16 (gray section) and XET_C (lined section). B showed conserved functional domains of AP2 (gray section). (DOC) [file pone.0094959.s001.doc]

A

1 GGG CTA TAA ATA CCC ACC CAT ATA TTC ATT TAC TTC TCG ATC ACC 45

46 TCA CAG AAA GTT CCA CTC TCA GTT CCC ATT TTG ATA TTC ACA ACA 90

91 CCA ACT AAA AAT AAT TTG AAA ATG GCT TCA GTT TTT TGT TCA AAT 135

M A S V F C S N 8

136 CAA ATA CAG CCG ATG ATA GCT CTC ATG GTT GGC GCA TTT TTG GTT 180

9 Q I Q P M I A L M V G A F L V 23

181 GCT TCA GCT GCT GGT AAC TTA AAC CAA GAT TTC GAC ATC ACT TGG 225

24 A S A A G N L N Q D F D I T W 38

226 GGC GAT GGC CGC GCC AAG ATT CTC AAC AGC GGA GAG CTT CTT ACG 270

39 G D G R A K I L N S G E L L T 53

271 CTT TCG CTT GAC AAG GCC TCG GGG TCG GGC TTC CAG TCC AAA AAA 315

54 L S L D K A S G S G F Q S K K 68

316 GAG TAT TTA TTT GGA AAG ATT GAT ATA CAG CTC AAG CTT GTC CCT 360

69 E Y L F G K I D I Q L K L V P 83

361 GGA AAC TCC GCC GGA ACT GTC ACT GCC TAT TAT TTG TCC TCA CAA 405

84 G N S A G T V T A Y Y L S S Q 98

406 GGT TCA ACC CAT GAT GAG ATA GAC TTT GAA TTC TTG GGA AAT TTG 450

99 G S T H D E I D F E F L G N L 113

451 AGC GGT GAT CCT TAC ATT CTG CAC ACT AAT ATC TTC AGC CAA GGC 495

114 S G D P Y I L H T N I F S Q G 128

496 AAG GGT AAC AGA GAG CAA CAA TTC TAC TTG TGG TTC AAC CCA ACT 540

129 K G N R E Q Q F Y L W F N P T 143

541 GCT GAT TTC CAC ACC TAT TCC ATC CTT TGG AAC CCC CAA CGC ATT 585

144 A D F H T Y S I L W N P Q R I 158

586 ATT TTT TCT GTG GAT GGC ACA CCC ATT AGA GAG TAT AAG AAT GCG 630

159 I F S V D G T P I R E Y K N A 173

631 GAG TCA ATC GGA GTT CCA TAC CCA AAG AAC CAG CCC ATG AGG CTA 675

174 E S I G V P Y P K N Q P M R L 188

676 TAC TCT AGC CTT TGG AAT GCG GAT GAT TGG GCT ACA AGG GGC GGG 720

189 Y S S L W N A D D W A T R G G 203

721 CTT GTC AAG ACA GAC TGG ACT CAA GCA CCC TTC ACT GCT TCT TAT 765

204 L V K T D W T Q A P F T A S Y 218

766 AGA AAC TTC AAT GCC GAT GCT TGT GTT CAG TCT TCT TCT GGG GCG 810

219 R N F N A D A C V Q S S S G A 233

811 TCT TCT TCT TCA TGC GCA AGC TCT AGC AAC ACT GCC AAC TGG TTC 855

234 S S S S C A S S S N T A N W F 248

856 TCG GAA GAA TTG GAC ACA ACA AGG CAG GAG AGG CTG AAA TGG GTG 900

249 S E E L D T T R Q E R L K W V 263

901 CAA AAC AAT TAC ATG ATC TAC AAT TAC TGC ACT GAC ATC AAG AGA 945

264 Q N N Y M I Y N Y C T D I K R 278

946 TTC CCT CAA GGC CTG CTA CCA GAA TGC AAC ATG TCT TAA AGA TAC 990

279 F P Q G L L P E C N M S *

991 AGA GCC CAT GTA TTG CAC ATT TTT TAA TAC TAT GAT TTT TTT TTA 1035

1036 TTC TAT ATG AAA TTC CAT TAT GTA ATT CTT TTG TAG AGA TTA ATA 1080

1081 AAA AAT AAT TTT AAG TTC GAC 1101

B

1 GAA AGG AGA GAG ATT AAA TTC TTC TTC TTC ATA TAT CTC TTA GTT 45

46 TCC TCC CCT CTT CTC CTA TCT CTT CTT GTT TAT GTA CCT CCA CCA 90

91 AGC ATA AAA TTT CAA GAT AAA TAC TCT CTA TTT GGT AGA TAG GGA 135

136 GGA TTT CAA TTG ATT GAT CAT TTG CAG CAG TGT AGT AAT CCT CAA 180

181 GAA GCC ATG AAT TCA AAC AAC TGG CTC TCC TTT CCT CTT TCT CCT 225

M N S N N W L S F P L S P 13

226 ACT CAT CCT TCT TTG CCT ACT CAT GAT CTT CGT GAG ACA TCT CAA 270

14 T H P S L P T H D L R E T S Q 28

271 TCC CAT CAT TTC TCT CTA GGG TTA GTG AGT GAC AAC ATC GAC AGT 315

29 S H H F S L G L V S D N I D S 43

316 CCG TTT CAA GAT CAA GAG TGG AAT TTG ATT AAT GTT CAA GGA AGC 360

44 P F Q D Q E W N L I N V Q G S 58

361 AAT GAG GTT CCA AAG ATA GCA GAC TTT CTT GGT GTG GGA AAA TCA 405

59 N E V P K I A D F L G V G K S 73

406 GAA AAC CAG ACT TCA GAT CTT GTT TAT AAT GAA ATC CAT GCA AAT 450

74 E N Q T S D L V Y N E I H A N 88

451 GAA TCC GAT TAC CTA TTC ACC AAC CAC AAC TTA TTG CCA GTG CCA 495

89 E S D Y L F T N H N L L P V P 103

496 AAC ACA TTA GCA GTG GCT CCT ACC GAT AAC TAT GAT CTT CCT GAA 540

104 N T L A V A P T D N Y D L P E 118

541 AAT GCC AGT AAT TTA CAA TCA TTA ACA TTG TCT ATG GGA AGT GGC 585

119 N A S N L Q S L T L S M G S G 133

586 AAG CGT TTG ACA TGT GAA ACT AGT GGT GAA AAT AAT AAT AGT ACC 630

134 K R L T C E T S G E N N N S T 148

631 ACG ACT GCC ATC GTT GAG GCT ACT CCA AGA AGA ACT TTG GAT ACG 675

149 T T A I V E A T P R R T L D T 163

676 TTT GGG CAA AGA ACA TCA ATT TAT CGC GGT GTA ACT AGG CAT AGA 720

164 F G Q R T S I Y R G V T R H R 178

721 TGG ACT GGA AGG TAT GAA GCT CAT TTA TGG GGT AAT AGT TGT AGA 765

179 W T G R Y E A H L W G N S C R 193

766 AGG GAA GGT CAA TCA AGG AAA GGT CGC CAA GTC TAC TTG GGT GGG 810

194 R E G Q S R K G R Q V Y L G G 208

811 TAT GAC AAA GAA GAG AAA GCA GCT AGG GCT TAT GAC ATG GCT GCA 855

209 Y D K E E K A A R A Y D M A A 223

856 CTG AAG TAC TGG GGA GCA TCT ACC ACT ACC AAT TTC CCA ATC ACT 900

224 L K Y W G A S T T T N F P I T 238

901 AAC TAT GAA AAG GAA CTT GAG GAT ATG AAA CAC ATG ACT AGA CAA 945

239 N Y E K E L E D M K H M T R Q 253

946 GAA TTT GTG GCC TCC ATT CGA AGG AAG AGT AGT GGC TTT TCA AGG 990

254 E F V A S I R R K S S G F S R 268

991 GGT GCA TCC ATG TAT AGG GGT GTT ACA AGG CAT CAT CAA CAT GGG 1035

269 G A S M Y R G V T R H H Q H G 283

1036 AGA TGG CAA GCA AGG ATT GGT AGA GTT GCT GGC AAC AAG GAT CTC 1080

284 R W Q A R I G R V A G N K D L 298

1081 TAT TTG GGA ACT TTC AGC ACA GAG GAA GAA GCA GCC GAG GCC TAC 1125

299 Y L G T F S T E E E A A E A Y 313

1126 GAC ATT GCA GCC ATA AAG TTC CGA GGC CTC AAC GCC GTC ACA AAC 1170

314 D I A A I K F R G L N A V T N 328

1171 TTC GAC ATG ACC CGT TAC GAC GTC AAG AGC ATC CTG GAA AGC AAC 1215

329 F D M T R Y D V K S I L E S N 343

1216 ACT TTA CCC ATC GGA GGC GGA GCC GCC AAA CGC CTA AAA GAG GCA 1260

344 T L P I G G G A A K R L K E A 358

1261 CAA GCT ATC GAA TCG TCG CGA AAA CGC GAT GAA ATG ATA GCT CTC 1305

359 Q A I E S S R K R D E M I A L 373

1306 GGG TCG GGC TTT CAA TAC GGG AAC CCG GTT CCA ACC CCA TTA CAA 1350

374 G S G F Q Y G N P V P T P L Q 388

1351 GCA TAC CCT CTA ATG CAA CAA CAG TAC GAG CAG CAA CAA ACA ACA 1395

389 A Y P L M Q Q Q Y E Q Q Q T T 403

1396 GTA GTA CCT TTA CTA ACC CTA CAA CAA AAC CCC GAG ATT TCG CAC 1440

404 V V P L L T L Q Q N P E I S H 418

1441 TTT TCC CAA GAC GCT CAA TTC CAC CAA AGT TAT ATA CAA ACT CAA 1485

419 F S Q D A Q F H Q S Y I Q T Q 433

1486 CTA CAA TTG AAT CAA CAG CAA CAG CAA CAA CAA TCT GTT GGG AAT 1530

434 L Q L N Q Q Q Q Q Q Q S V G N 448

1531 CAT CAG TTC TAT AAT AGT TAC CTT CAG AAT AAT CCG GGT TTT TTG 1575

449 H Q F Y N S Y L Q N N P G F L 463

1576 CAT GGG TTT ATG GGC ATG GGC GGG TCT TCA TCC TCG GTT ATG GAT 1620

464 H G F M G M G G S S S S V M D 478

1621 AAT AAT AAC AAT GGG AGT TCA AGT GGT GGG AGT TAT AGT GGA GGG 1665

479 N N N N G S S S G G S Y S G G 493

1666 TAT TTA GGC AAT TCC ATG TCC GGG AAC GGC GGA GGT TCG TCG GTG 1710

494 Y L G N S M S G N G G G S S V 508

1711 GAG GAG CTT GGG ATG GTG AAA GTG GAT TAT GAT ATG CCC GCC GGG 1755

509 E E L G M V K V D Y D M P A G 523

1756 AGT TAT GGT GGA TGG TCC GGG GAT TCG TCG GGT CAA GTA TCA AAT 1800

524 S Y G G W S G D S S G Q V S N 538

1801 CCG GGC ATG TTT TCA ATG TGG AAT GAC TGA GAT TTT TGA AGA ATG 1845

539 P G M F S M W N D *

1846 GGG TGA ATG GGG AAA ATG GGT TTA GTT GCA TTA TTT GCA TGG TGA 1890

1891 AGT TTA GGT GCT TAT TTT GGT TTT GAC TTC TGA CAA TGT CTT TCA 1935

1936 TTT AAT TAA TAG GAT GAG AGG GTA TGA CAA AGA ACT AAT TTG GTC 1980

1981 AGC CTA TAT GTT TCT TTT TAT TGT TAT TAA GAG CAG TTG ATT ATC 2025

**Figure S1. The gene and amino acid sequences of *EsXTH1* and *EsPLT1* from Siberian ginseng.** **A** showed conserved functional domains of Glyco_hydro_16 (*gray section*) and XET_C (*lined section*). **B** showed conserved functional domains of AP2 (*gray section*).
